# Safety and efficacy of subtotal or total parathyroidectomy for patients with secondary or tertiary hyperparathyroidism in four academic centers in the Netherlands

**DOI:** 10.1007/s00423-018-1726-6

**Published:** 2018-11-10

**Authors:** Willemijn Y. van der Plas, Rorderick R. Dulfer, Ezra Y. Koh, Liffert Vogt, Natasha M. Appelman-Dijkstra, Abbey Schepers, Joris I. Rotmans, Robert A. Pol, Tessa M. van Ginhoven, Ewout J. Hoorn, Els J. M. Nieveen van Dijkum, Anton F. Engelsman, Martin H. de Borst, Schelto Kruijff, on behalf of the Dutch Hyperparathyroidism Study Group (DHSG)

**Affiliations:** 1 Department of Surgery, University Medical Center Groningen, University of Groningen, Hanzeplein 1, 9700 RB Groningen, The Netherlands; 2 Department of Surgery, Erasmus Medical Center, Erasmus University Rotteredam, Rotterdam, The Netherlands; 3 Department of Surgery, Amsterdam UMC, University of Amsterdam, Amsterdam, The Netherlands; 4 Department of Nephrology, Amsterdam UMC, University of Amsterdam, Amsterdam, The Netherlands; 5 Department of Endocrinology, Leiden University Medical Center, Leiden University, Leiden, The Netherlands; 6 Department of Surgery, Leiden University Medical Center, Leiden University, Leiden, The Netherlands; 7 Department of Internal Medicine, Leiden University Medical Center, Leiden University, Leiden, The Netherlands; 8 Department of Internal Medicine, Division of Nephrology and Transplantation, Erasmus Medical Center, Rotterdam, The Netherlands; 9 Department of Nephrology, University Medical Center Groningen, University of Groningen, Groningen, The Netherlands

**Keywords:** Parathyroidectomy, End-stage renal disease, Outcomes, Hyperparathyroidism

## Abstract

**Purpose:**

Hyperparathyroidism (HPT) is a common abnormality in patients with end-stage renal disease (ESRD). Since the introduction of cinacalcet in 2004, a shift from surgery toward predominantly medical treatment has occurred. Surgery is thought to be associated with more complications than oral medication. The aim of this retrospective study was to evaluate 30-day outcomes and effectiveness of parathyroidectomy (PTx) in ESRD patients in the Netherlands.

**Methods:**

A national database containing data from four academic medical centers in the Netherlands of patients with ESRD-related HPT, who had undergone PTx and kidney transplantation between 1994 and 2015, was established. Primary endpoints were 30-day mortality and complication rate. Secondary endpoints were biochemical measurements.

**Results:**

We identified 187 HPT patients undergoing PTx, with a median age of 46 years. Median preoperative PTH level was 866 pg/mL (interquartile range [IQR] 407–1547 pg/mL). At 3 months, the median PTH drop from baseline was 93% (IQR, 71–98%) to a median of 61 pg/mL (IQR, 23–148 pg/mL, *p* < 0.001). Over the 25-year inclusion period, 13 patients (7.0%) required re-exploration for persistent or recurrent disease. Thirty-day mortality and complication rate were 0.0% and 7.9% respectively. Median serum calcium levels improved significantly postoperatively from 2.6 (2.4–2.8) mmol/L to 2.3 (2.1–2.5) mmol/L (*p* < 0.001).

**Conclusions:**

PTx is a safe and effective procedure in the frail ESRD population. These data show that there should be no reluctance for surgical intervention and when indicated, nephrologists can safely refer these patients for PTx.

## Introduction

Hyperparathyroidism (HPT), both secondary and tertiary, is a common complication with a prevalence up to 30–49% in patients with end-stage renal disease (ESRD) [1, 2]. ESRD-related HPT has been associated with severe bone disorders, cardiovascular complications, and increased mortality [3–6]. More than a decade ago, the treatment algorithm of HPT consisted of calcium salts, vitamin D sterols, and (sub)total parathyroidectomy (PTx). Since its introduction in 2004, the calcimimetic agent cinacalcet is being used to treat patients with HPT, when vitamin D analogs and phosphate binders are insufficient [7, 8]. The latest update of the Kidney Disease Improving Global Outcomes (KDIGO) Chronic Kidney Disease—Mineral and Bone Disorder (CKD-MBD) guideline recommends cinacalcet even as a first step option together with vitamin D and phosphate binders in patients with secondary HPT [8]. Despite the lack of randomized studies that compare cinacalcet with surgical treatment, the advent of cinacalcet, and in parallel, an increasing perception of PTx as a high-risk procedure in this fragile ESRD population has, among other reasons, led to a shift from surgery toward predominantly medical treatment [2, 9]. Several studies have shown a reduction in the number of parathyroidectomies per year since the introduction of cinacalcet [10, 11]. In a previous study, we reported that the introduction of cinacalcet is associated with a 2-year delay of surgery in which patients still had continuously elevated parathormone (PTH) levels [12]. On the other hand, the inability of cinacalcet to achieve an efficient and persistent lowering of PTH levels has been reported in multiple studies [13, 14]. Moreover, cinacalcet use can be accompanied by serious adverse events, such as vomiting and diarrhea, which potentially leads to discontinuation of the drug [14].

In recent years, parathyroid surgery has undergone a great evolution with the use of minimally invasive techniques and heat-sealing devices [15, 16]. However, surgery is still thought to be associated with more complications than oral medication. A nationwide study using United States Renal Data System (USRD) ESRD data evaluating the outcome of 4435 hemodialysis patients undergoing PTx concluded that parathyroid surgery is associated with significant morbidity [17]. However, evidence on the perioperative risk of PTx performed in specialized, high-volume centers is poorly documented and the question is whether the hesitancy for surgical referral also applies to such centers. Therefore, the aim of this study was to evaluate 30-day outcomes and effectiveness of PTx in ESRD patients in the Netherlands. The Dutch Hyperparathyroidism Study Group was established in 2016 in the Netherlands to address, among others, this issue.

## Material and methods

### Study population

A national retrospective database of patients with ESRD-related HPT who underwent both PTx and kidney transplantation (KTx) in the Netherlands was established, to answer all distinct research questions. Data from four academic medical centers (Academic Medical Center [AMC], Erasmus Medical Center [EMC], Leiden University Medical Center [LUMC], and University Medical Center Groningen [UMCG]) of patients with ESRD-related HPT undergoing PTx between 1994 and 2015 were extracted. Databases were cross-checked between participating centers. The study population included all patients diagnosed with ESRD in the aforementioned medical centers, aged ≥ 18 years who underwent PTx and KTx. Secondary HPT was defined as HPT in patients receiving hemodialysis and tertiary HPT as HPT in patients who underwent KTx. Approval from the medical ethical committee boards of all centers was retrieved. Data was collected and stored in concordance with the Declaration of Helsinki.

### Patient characteristics and primary and secondary endpoints

Patient characteristics were derived from each hospital’s electronic medical record system. Collected data consisted of age, sex, primary ESRD cause, time on dialysis, history of KTx, preoperative American Society of Anesthesiologists (ASA) physical status classification, type of PTx, medication related to calcium-phosphate metabolism, postoperative complications (including recurrent laryngeal nerve damage, wound problems, hospital-acquired pneumonia, and intensive care unit admission), and mortality. Patients who presented with hoarseness pre- or postoperatively were offered laryngoscopy performed by a qualified ear nose and throat specialist to evaluate the vocal cords and intactness of the recurrent laryngeal nerve. The primary outcome was 30-day mortality and complication rate (including the aforementioned complications). Secondary endpoints were biochemical measurements, including pre- and postoperative PTH levels, calcium, phosphate, albumin, alkaline phosphatase (ALP), and creatinine. Furthermore, reoperation rate and time until reoperation were recorded. Serum calcium levels were adjusted for albumin using the following equation: adjusted total calcium (mmol/L) = measured calcium (mmol/L) + (0.025 × (40 − [albumin (g/L)]). Reference interval was 2.20–2.60 mmol/L. To calculate the proportion of patients that were hypocalcaemic during follow-up, serum-adjusted calcium concentrations were categorized into three groups: low calcium (< 2.20 mmol/L), adequate calcium (2.20–2.60 mmol/L), and high calcium (> 2.6 mmol/L). Postoperative hypocalcaemia rates were compared between patients who underwent subtotal PTx and total PTx with AT. The reference interval for serum phosphate levels was 0.7–1.5 mmol/L. In a subanalysis, we compared all biochemical levels between patients with secondary HPT and those with tertiary HPT.

### Surgical protocol

Patients were referred for surgical treatment in case of disease refractory to pharmacological treatment (including vitamin D analogs, phosphate binders, and calcimimetics). Other indications for surgical referral included intolerance to or noncompliance of cinacalcet, severe disease with highly elevated serum PTH and calcium levels, symptomatic disease, patient’s preference, and persistent hypercalcemia after KTx. All patients underwent a subtotal PTx or total PTx + autotransplantation (AT). In subtotal PTx, 3.5 of the four glands were resected. Total PTx + AT comprised of the resection of all four parathyroid glands whereafter half of the most normal-appearing parathyroid gland was minced and then autotransplanted in the sternocleidomastoid muscle or into subcutaneous pockets of the forearm. During PTx, intraoperative PTH measurements were available in all centers. When intraoperative PTH measurement was used, serum concentration PTH was determined before resection of the parathyroid glands, and 5, 10, 15, and 20 min after resection. In general, an intraoperative drop of PTH of ≥ 85% was considered to be sufficient for a successful operation [18]. In case of an inadequate PTH drop, surgeons looked for extra or ectopic parathyroid glands.

In each medical center, pre- and postoperative calcium management was handled according to the local protocol and included both oral and, if necessary, intravenous calcium supplementation.

### Statistical analysis

Statistics were performed using SPSS Statistics version 24.0 (IBM Corporation, Armonk, NY, USA.). Continuous variables are described as mean ± SD (normal distribution) or median with interquartile range (IQR) in case of skewed distribution. Distribution was assessed with the Shapiro-Wilk normality test. Categorical variables are expressed as number (*n*) and percentage (%). Subsequent laboratory values were compared using the paired sample *t* test or related sample Wilcoxon signed-rank test. *p* values ≤ 0.05 were considered statistically significant.

## Results

### Patient characteristics

**Fig. 1:**
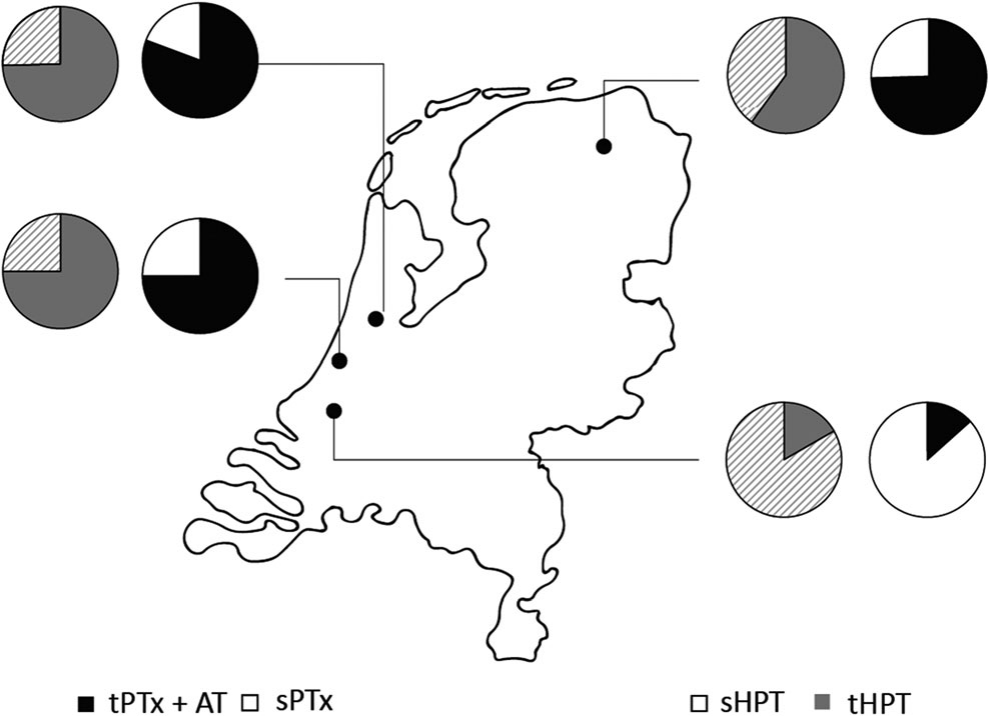
Type of HPT and PTx per medical center

A total of 187 patients were included in this study. Baseline characteristics of the included patients are listed in Table 1. Median age was 46 years (IQR, 33–57), and 50.3% were female. Of all patients, 58.3% underwent total PTx + autotransplantation (AT) whereas 41.7% underwent subtotal PTx. Sixty-nine percent of patients were classified ASA III or higher. Before surgery, 70.7% of patients were using phosphate binders, 61.9% calcium supplements, and 77.1% vitamin D analogs. Eighteen percent used calcimimetics. Of all patients, 103 (55%) underwent a KTx after PTx; the remaining 45% underwent a KTx prior to PTx. Median time until KTx was 22 (IQR, 10–38) months after PTx. The proportion of secondary (patients on dialysis) and tertiary (patients with a kidney transplant) HPT patients and type of PTx per center are shown in Fig. 1.

**Table 1:** Characteristics of ESRD patients at the time of PTx

	*N* = 187
Age, y (IQR)	46 (33–57)
Female sex, *n* (%)	94 (50.3)
Diabetes mellitus, *n* (%)	
DM type 1	7 (3.7)
DM type 2	18 (9.6)
Type renal replacement therapy, n (%)	
None	13 (7.0)
Hemodialysis	49 (26.2)
Peritoneal dialysis	41 (21.9)
Kidney transplantation	84 (44.9)
Type of PTx, *n* (%)	
Total PTx + AT	109 (58.3)
Subtotal PTx	78 (41.7)
ASA classification, *n* (%)	
ASA II	58 (31.0)
ASA III	126 (67.4)
ASA IV	3 (1.6)

*KTx*, kidney transplantation; *DM*, diabetes mellitus; *PTx*, parathyroidectomy; *AT*, autotransplantation; *ASA*, American Society of Anesthesiologists

### Preoperative and short-term laboratory values

Preoperative biochemical levels are shown in Table 2, separated for patients with secondary hyperparathyroidism (e.g., those who did not have a KTx in medical history) and patients with tertiary hyperparathyroidism (e.g., those who underwent KTx prior to PTx). All preoperative laboratory values were significantly different between the two groups. Median preoperative PTH level of all patients was 866 pg/mL. Intraoperative PTH measurements were available in 120 patients. During PTx, PTH levels dropped significantly during surgery with a median of 86% (IQR, 72–95%, *p* < 0.001). Median drop of PTH of all patients after 3 months was 93% (IQR, 71–98%) from 866 (407–1447) pg/mL to 61 (23–148) pg/mL. Postoperatively, PTH levels were not significantly different for patients with secondary HPT compared to tertiary HPT patients.

**Table 2:** Laboratory values preoperatively

	Total	Secondary hyperparathyroidism (PTx before KTx)	Tertiary hyperparathyroidism (PTx after KTx)	*p* value
PTH, pg/mL	866 (407–1547)	1143 (702–1776)	476 (243–1162)	< 0.01
Adjusted calcium, mmol/L	2.6 (2.4–2.8)	2.5 (2.3–2.7)	2.7 (2.5–2.9)	< 0.01
Phosphate, mmol/L	1.4 (0.9–1.9)	1.77 (1.5–2.1)	0.90 (0.7–1.35)	< 0.01
Creatinine, μmol/L	477 (137–927)	828 (523–1018)	141 (112–426)	< 0.01

*PTx*, parathyroidectomy; *KTX*, kidney transplantation; *PTH*, parathyroid hormone

Median-adjusted calcium levels improved significantly after PTx from a median of 2.6 (2.4–2.8) mmol/L to 2.3 (2.1–2.5) mmol/L (*p* < 0.001) and did not significantly differ between secondary HPT patients and tertiary HPT patients.

Preoperative phosphate levels were high in patients with secondary HPT and low but within the range of normal (0.81–1.45 mmol/L) in patients with tertiary HPT (Table 2). Of all patients, 56.7% of patients had serum phosphate concentrations above the upper limit of normal, mainly reflecting secondary HPT patients. Median phosphate levels did not change significantly after surgery (*p* = 0.1); at 3 months of follow-up, 43.9% of patients had serum phosphate levels above the upper limit of normal. Postoperative phosphate levels were significantly different at 3, 6, and 12 months between secondary and tertiary HPT levels, but all within the reference range.

### Long-term laboratory values

**Fig. 2:**
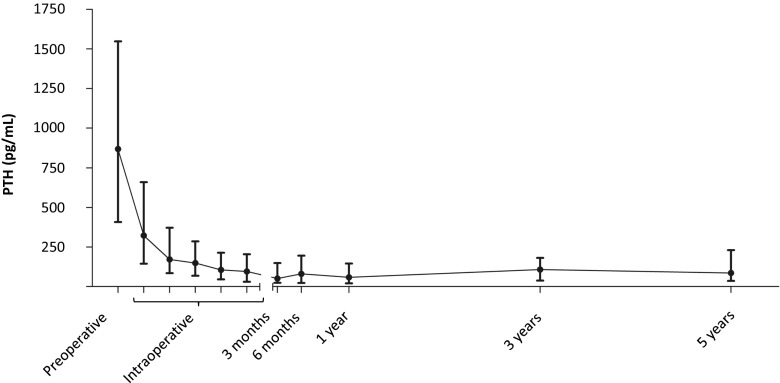
Parathormone (PTH) levels over time

Postoperative serum PTH levels remained low and overall did not increase during the 5-year follow-up compared to the levels of 3 months postoperatively (Fig. 2 and Table 3). At 3 months, 38.7% were hypocalcaemic (adjusted serum calcium levels < 2.20 mmol/l). Fourteen patients (9.9%) still had calcium levels above the upper limit of normal. The proportion of patients within the reference range at 5 years postoperatively was 68.3%. Median corrected calcium levels remained within the reference interval (2.20–2.60 mmol/L) and did not increase significantly during follow-up. All postoperative laboratory values are displayed in Table 3.

**Table 3:** Postoperative laboratory values

	3 months	6 months	1 year	3 years	5 years
PTH, pg/mL	61 (23–148)	80 (23–195)	58 (22–143)	106 (38–181)	85 (35–230)
Adjusted calcium, mmol/L	2.3 (2.1–2.5)	2.3 (2.1–2.4)	2.3 (2.1–2.4)	2.3 (2.2–2.4)	2.3 (2.2–2.4)
Phosphate, mmol/L	1.2 (0.9–1.6)	1.3 (0.9–1.7)	1.2 (0.9–1.7)	1.1 (0.9–1.4)	1.0 (0.9–1.3)
Creatinine, μmol/L	545 (148–1018)	476 (150–995)	271 (142–1001)	183 (123–601)	167 (116–224)

*PTH*, parathormone

### Postoperative complications

Postoperative complications are listed in Table 4. Of all patients who underwent PTx, the 30-day mortality rate was zero. Four patients experienced temporary hoarseness after surgery, which was objectified by laryngoscopy. Clinically, all four patients had full recovery of their voice within 1 year. In 48.5% of the patients, hypocalcaemia was reported during hospital admission. Intensive care admission to treat hypocalcaemia was required in 2.4% of the patients. Three months after PTx, 37.4% of patients were still using calcium supplements. Calcium supplements were required in 32.6% of patients at 1 year after surgery. No difference in postoperative hypocalcaemia rates was found between patients who underwent subtotal PTx and those who underwent total PTx with AT at any time point after PTx.

**Table 4:** Thirty-day mortality and morbidity, *n* (%)

Mortality	0 (0.0)
Morbidity	13 (7.9)
Temporary recurrent laryngeal nerve paralysis	4 (2.4)
Surgical site problems^†^	3 (1.8)
Hospital-acquired pneumonia	2 (1.2)
ICU admission	4 (2.4)

^†^Including wound infection and hemorrhage

### Reoperation rates

Of all 187 patients, 13 patients (7.0%) underwent a reoperation for persistent or recurrent disease. Median time until re-exploration was 6 months (IQR, 1–24 months). One patient (0.5%) underwent re-exploration during the initial admission for a postsurgical hemorrhage.

## Discussion

End-stage renal disease (ESRD) is often complicated by the development of hyperparathyroidism (HPT); more than 80% of patients with a glomerular filtration rate (GFR) below 20 mL/min develop serum PTH levels exceeding the upper limit of normal [19]. Historically, patients were referred for parathyroidectomy (PTx) when vitamin D analogs and phosphate binders were no longer sufficient to control calcium-phosphate homeostasis [7]. However, since its introduction in 2004, cinacalcet has become the second step in the treatment of HPT and surgery rates decreased without strong evidence to support this practice [8, 20]. This multicenter retrospective study reports surgical outcomes of parathyroidectomy (PTx) in patients with ESRD-related HPT. Our results indicate that PTx is associated with no 30-day mortality and very low 30-day morbidity rates. Three months after surgery, serum PTH levels dropped significantly to a median of 61 pg/mL (93% drop) and remained low during 5 years of follow-up.

Our results are supported by multiple previous studies that reported high success rates of PTx [21, 22]. Previously documented PTH drop rates ranged from 89 to 98% [23–25]. Since the introduction of calcimimetics in 2004 however, PTx is thought to be associated with more complications than oral medication, and a paradigm shift in referring patients for surgery occurred [12]. On top of that, as mentioned before, authors of a nationwide study in the USA reported high complication rates following parathyroid surgery [17]. The authors observed a rehospitalization rate due to complications of 23.8% and almost 30% of these patients required ICU admission. Indications for ICU admission were not elaborated in the article. These findings are in sharp contrast with our results. This variance can be explained by the inclusion of Medicare insurance centers in this American study, which may have led to the inclusion of patients in low-volume, less specialized centers. The discrepancy between these results emphasizes the need to refer these patients to experienced medical centers dedicated to (para)thyroid surgery. In the Netherlands, surgeons, endocrinologists, and nephrologists work in close collaboration to improve patient health care; patients are elaborated preoperatively in a multidisciplinary meeting, surgeons are required to perform over 20 (para)thyroid surgeries a year, and after PTx, the patient is monitored by both their surgeon as well as their nephrologist/endocrinologist. A minimum number of required (para)thyroid procedures per surgeon ensure quality across medical centers. More in concordance with our results, multiple other studies also reported low complication rates after PTx [26, 27]. In the literature, HPT recurrence rates requiring reoperation ranged between 5 and 30%. Those results are in line with our findings (7%) [24, 28].

Hypocalcaemia is a frequently mentioned consequence of PTx in patients with secondary or tertiary hyperparathyroidism [29]. The finding that a substantial proportion of patients are temporarily hypocalcaemic after PTx emphasizes the need for strict observation and protocol in collaboration with a nephrologist or endocrinologist.

In the treatment of secondary and tertiary HPT, both pharmacological and surgical management are well-recognized options. Since its introduction of calcimimetics in 2004, cinacalcet gained a dominant position in the treatment algorithm and prescription patterns increased significantly over the years [19]. However, in 2012, the EVOLVE Trial Investigators published their results of a randomized controlled trial evaluating the effect of cinacalcet on the risk of death or cardiovascular events compared to placebo in 3883 dialysis patients [14]. The authors concluded that cinacalcet did improve serum PTH and calcium levels, but it did not significantly reduce the risk of death or cardiovascular events (cinacalcet vs. placebo, relative hazard 0.93, 95% confidence interval 0.85–1.02; *p* = 0.11). Of note, a significant difference of 1 year in age between the two groups, a high drug discontinuation rate, and a high cross-over between the two treatment arms may have led to the minimal impact of cinacalcet on the primary composite endpoint. Moreover, the use of cinacalcet is often accompanied with a range of (gastro-intestinal) adverse effects, leading to discontinuation or noncompliance [13, 14]. Almost half of the patients on cinacalcet participating in the EVOLVE trial reported adverse events compared to 19% of the placebo group.

These findings, together with the results of an increasing number of articles reporting on the limited impact of cinacalcet on PTH, its costs, side effects, and minimal beneficial impact on QoL should be taken into account when new guidelines are established in the future [13, 14, 30, 31]. A decisive randomized controlled study comparing pharmaceutical treatment with PTx head-to-head is still not available. This RCT is needed before the optimal treatment for these patients can be determined.

This study has several limitations that need to be addressed. First, the retrospective nature of this analysis could have led to selection bias in our recorded data and missing data was sometimes inevitable. For instance, patients were only included if they underwent both a PTx and KTx. Patients who underwent KTx before PTx as well as patients who underwent KTx after PTx were included. This may also have led to selection bias since patients who were not eligible for KTx were not included. Our conclusions of the safety of PTx might only be applicable in patients who are found to be also eligible for KTx. Secondly, this study population is a heterogeneous patient group with multiple comorbidities [32].

More than half of our patients underwent KTx after PTx after a median time of 22 months. This event influenced the biochemical results during long-term follow-up. One of the strengths of this multicenter study is that data from four academic hospitals in the Netherlands were included. Our findings can be generalized to other (academic) medical centers with a similar type of health care and quality standards.

In patients with secondary HPT, KTx leads to an amelioration of the disturbance of the calcium-phosphate homeostasis in more than half of the patients [33]. Therefore, if kidney transplantation is not expected in the near future, and in case of severe disease with debilitating symptoms, PTx is a safe and effective option. In patients with tertiary hyperparathyroidism, kidney transplantation apparently did not improve calcium-phosphate homeostasis and PTH levels continue to rise. In these patients, PTx is considered the only definite option to treat the persistent hyperparathyroidism.

In conclusion, our results indicate that PTx is a very safe and effective procedure in patients with ESRD-related HPT, a patient population with extensive comorbidities. Although an invasive procedure requiring general anesthesia, recovery is fast and without major complications. Our patients were operated in high-volume and high-experienced medical centers, which may have contributed to high success rates. Nevertheless, these findings implicate that there should be no reluctance in referring these patients for PTx, when indicated, despite their comorbidity and high ASA classification.
